# An Integrated Decentralised–Centralised Oncology Care Model to Improve Cancer Screening, Access, and Continuity of Care in Rural Eastern Cape, South Africa: Implementation Study at Nelson Mandela Academic Hospital

**DOI:** 10.3390/ijerph23081079

**Published:** 2026-08-19

**Authors:** Zukiswa Jafta, Muamabangu Jean Paul Milambo, Eric Maimela, Constance Rufaro Sewani-Rusike, Wilson Wezile Chitha

**Affiliations:** 1 Cancer Research & Innovation Platform of the Eastern Cape (CRIPEC), Division of Oncology, Walter Sisulu University, Mthatha 5100, Eastern Cape, South Africa; 2 Walter Sisulu Institute for Clinical Governance and Healthcare Administration, School of Public Health, Faculty of Medicine and Health Sciences, Walter Sisulu University, Mthatha 5100, Eastern Cape, South Africa; emaimela@wsu.ac.za (E.M.);; 3 Physiology Department, Faculty of Medicine and Health Sciences, Walter Sisulu University, Mthatha 5100, Eastern Cape, South Africa; crusike@wsu.ac.za

**Keywords:** cancer care delivery, decentralised oncology, rural health systems, implementation science, patient navigation, health systems strengthening, cancer screening, task shifting, low-resource settings, South Africa

## Abstract

**Highlights:**

**Public health relevance—How does this work relate to a public health issue?**

- This study addresses inequitable access to cancer care in rural, resource-constrained settings through evaluation of a decentralised–centralised service-delivery model.
- It examines health system barriers to timely cancer screening, diagnosis, and treatment in a high-burden, underserved population in South Africa.

**Public health significance—Why is this work of significance to public health?**

- The findings demonstrate that decentralisation can significantly increase cancer service utilisation and improve access to care in low-resource settings.
- The study provides empirical evidence on how integrated care models strengthen continuity of care and service integration across the cancer care continuum.

**Public health implications—What are the key implications or messages for practitioners, policy makers, and/or researchers in public health?**

- Decentralised screening and navigation support can reduce travel burden, improve early detection, and enhance patient retention in care.
- Scaling similar hybrid oncology models, alongside investment in workforce capacity and diagnostic pathways, can strengthen cancer care systems in comparable settings.

**Abstract:**

**Background:** Rural and resource-constrained settings face major barriers to timely cancer screening, diagnosis, and treatment due to limited specialist availability and centralised service-delivery models. In the Eastern Cape, a largely rural province with a constrained oncology workforce, a decentralised–centralised hybrid model was introduced to improve access to cancer care. Nelson Mandela Academic Hospital serves as the central referral hub within this model. This study evaluates the implementation process and impact of this decentralised cancer care model on service utilisation, access, and continuity of care. **Methods:** A quantitative quasi-experimental pre–post implementation and quality improvement evaluation was conducted using retrospectively collected routine service utilisation and programme data from April 2023 to February 2025. The study assessed the impact of a decentralised oncology care model on access, service integration, and utilisation outcomes. Data from facility registers and district health information systems were managed using Microsoft Excel and analysed using Stata and IBM SPSS Statistics. Descriptive statistics, correlation analysis, and linear regression were used to compare pre- and post-implementation changes in patient volumes, screening coverage, referral completion, workforce capacity, gender distribution, and service uptake. The intervention decentralised screening, diagnosis, follow-up, and patient navigation services to district and satellite facilities while centralising specialised oncology care at referral centres to improve accessibility, efficiency, and continuity of care. **Results:** Cancer patient attendance increased substantially over the study period, from 355 patients in April 2023 to a peak of 1039 in April 2024, with a sustained upward trend (B = 18.03, *p* = 0.005), reflecting an average monthly increase of 18 patients. Female patients accounted for most visits, while male attendance showed a significant increasing trend (B = 8.03, *p* < 0.001). Service integration improved, with strong positive correlations between new patient registrations, follow-up care, palliative services, and inpatient admissions, indicating an expanding continuum of care. Breast and cervical cancers contributed the highest service burden, while cervical and lung cancers showed significant upward trends. Seasonal variation in attendance was observed, particularly during festive periods. From an implementation perspective, screening coverage for priority cancers increased by 18%, while 732,349 individuals were reached through community awareness initiatives. Access improved substantially, evidenced by a reduction of 56,400 km in cumulative patient travel distance over one year. Workforce capacity was strengthened through the training of 517 healthcare workers, and 1943 patients received structured navigation support. Referral efficiency and continuity of care improved, although persistent bottlenecks were observed in diagnostic and referral pathways. **Conclusions:** The decentralised–centralised oncology care model demonstrated improved cancer service utilisation, access, and continuity of care in a rural, resource-limited setting. However, increasing patient volumes and interconnected service demands place additional pressure on health system capacity. Sustained investment in workforce development, screening—particularly for cervical cancer—and system efficiency is required. This model provides a scalable and context-appropriate framework for strengthening oncology services in similar low-resource settings.

## 1. Background

Cancer remains a leading cause of morbidity and mortality globally, with approximately 20 million new cases and nearly 10 million deaths annually, reflecting a growing public health burden [1,2]. Despite advances in prevention, early detection, and treatment, substantial disparities persist between high-income countries and low- and middle-income countries (LMICs), driven by inequities in health system capacity, workforce distribution, and access to specialised oncology services [1,3]. Over 70% of global cancer deaths occur in LMICs, where late-stage presentation and limited treatment access remain common [1,3].

In high-income settings, cancer outcomes have improved through structured, largely centralised models of care supported by organised screening and multidisciplinary oncology services. In the United States, coordinated programmes led by the American Cancer Society and American Society of Clinical Oncology have strengthened early detection and survival outcomes, although inequities persist among rural and underserved populations [4,5]. Similarly, many European systems rely on centralised cancer centres supported by national screening programmes, which improve outcomes but still show regional disparities in access to radiotherapy and specialist care [2]. These models reflect a predominantly centralised “hub-based” approach to oncology service delivery.

In contrast, many LMICs in Asia and sub-Saharan Africa face fragmented and under-resourced oncology systems characterised by late diagnosis, weak referral pathways, and limited specialist availability [2]. In Africa, the rising burden of breast, cervical, and prostate cancers is compounded by severe workforce shortages and inadequate diagnostic infrastructure, resulting in delayed presentation and poor outcomes [2]. South Africa reflects this dual burden, with advanced tertiary oncology services in urban centres coexisting alongside significant rural inequities, particularly in provinces such as the Eastern Cape, where access to care remains highly constrained [6,7,8].

The Eastern Cape exemplifies these challenges, with Nelson Mandela Academic Hospital (NMAH) functioning as the primary oncology referral hub for a largely rural population. Patients frequently experience long travel distances, delayed referrals, and fragmented care pathways, exacerbated by severe oncology workforce shortages (approximately one specialist per three million population) and under-resourced district-level facilities. These system constraints contribute to late diagnosis, poor continuity of care, and high patient access burdens.

**Figure 1:**
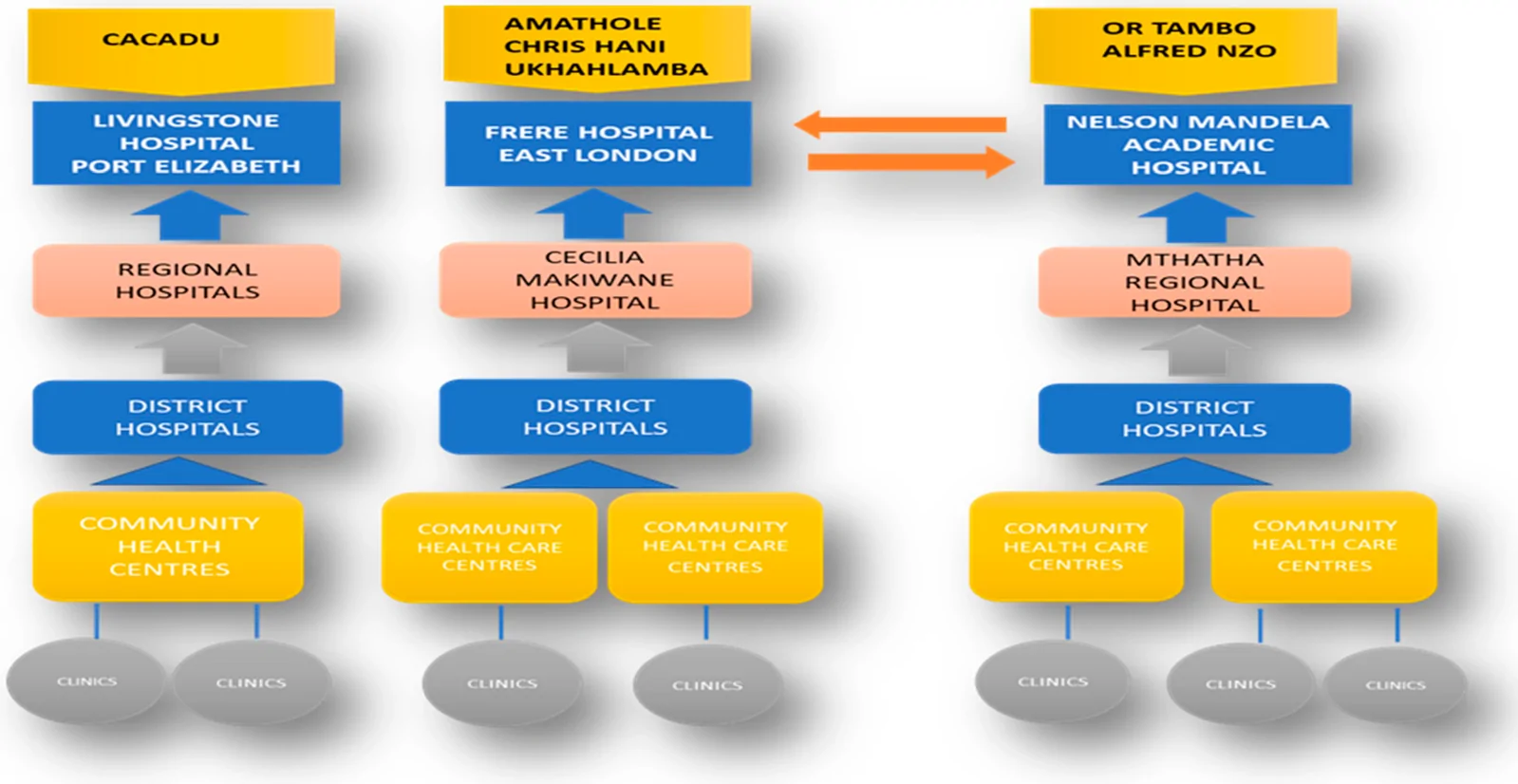
Hub-and-spoke hybrid or initial oncology care model.

The previous (centralised) model of oncology care in the province was highly concentrated at a single tertiary facility, where most services—including diagnosis, treatment, and follow-up—were delivered at the provincial referral hospital (Figure 1). This structure created significant access barriers for rural populations due to long travel distances, high transport costs, and limited continuity of care. It also placed excessive pressure on a severely constrained specialist workforce (approximately one oncologist per three million people), contributing to delayed diagnosis, treatment bottlenecks, and fragmented patient pathways [9,10,11,12,13,14]. As a result, many patients presented at late stages of disease, reflecting both weak community awareness and limited early detection capacity within primary health care services [10,12].

The proposed model [10] selected oncology services to district and satellite facilities while retaining high-complexity care at the central hub, Nelson Mandela Academic Hospital (NMAH) (Figure 1). Under this system, screening, early diagnosis, follow-up, survivorship care, and selected low-risk treatments are delivered closer to communities, supported by task-sharing, telemedicine, training, and bidirectional referral pathways [8,9]. The central hub remains responsible for complex diagnostics, chemotherapy, radiotherapy, and inpatient oncology care, while also providing oversight, mentorship, and down-referral of stable patients to peripheral sites. This integrated structure aims to improve access, reduce patient burden, strengthen continuity of care, and improve system efficiency in a resource-limited rural setting [9,11,15].

**Figure 2:**
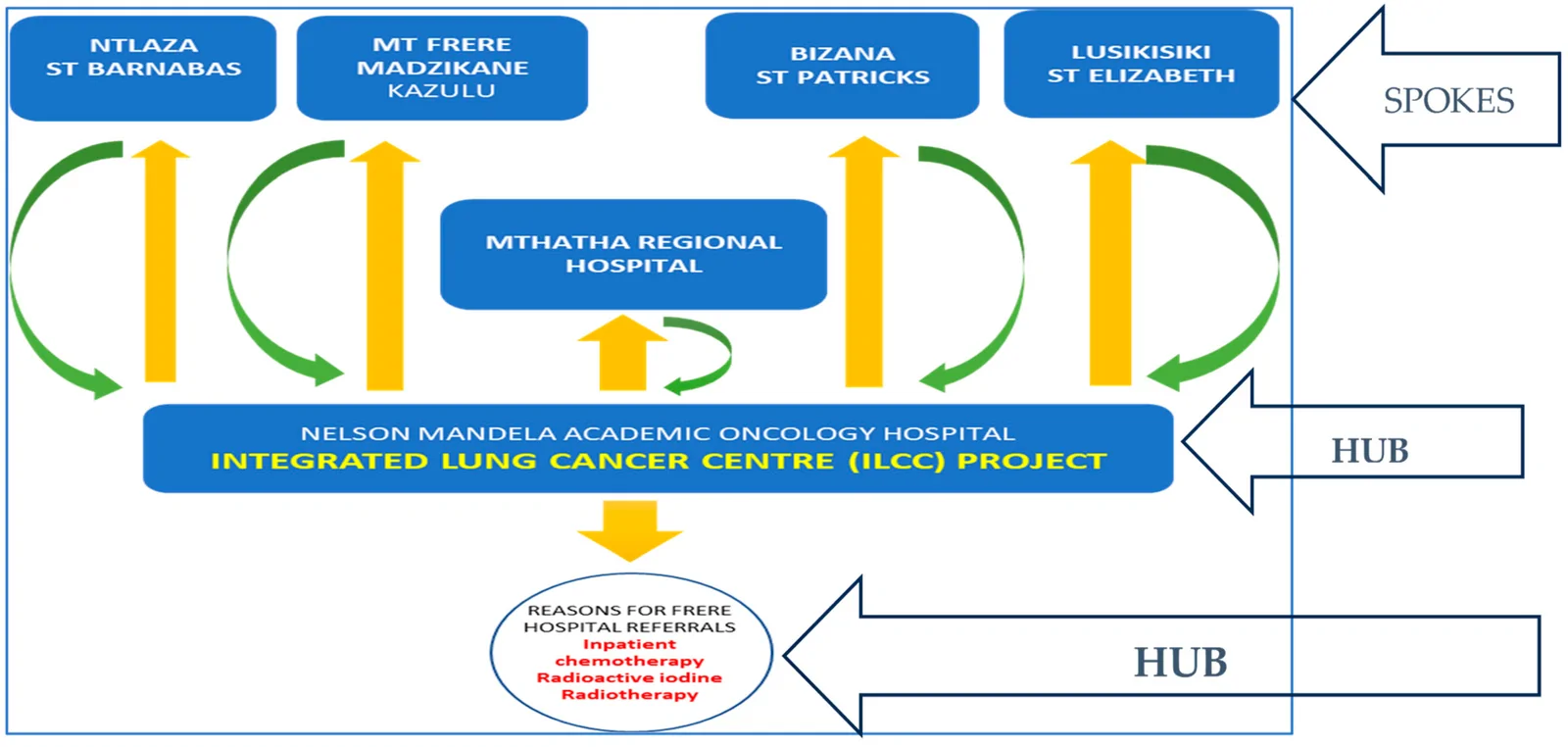
NMAH will serve as the hub for advanced cancer care and will receive and down refer patients to satellite cancer care sites (selected district hospitals).

This study is driven by interconnected patient- and health system-level constraints that limit equitable cancer care (Figure 2). Patient barriers include late presentation due to low health literacy, poor awareness of cancer symptoms, and significant socioeconomic challenges such as transport costs and geographic isolation [9,11,13]. Health system constraints include severe specialist shortages, under-resourced primary health care facilities, and weak diagnostic and referral capacity in rural districts [10,11,14]. In response, the decentralised–centralised model was developed as a quality improvement initiative to improve equity, strengthen early detection, enhance continuity of care, and optimise use of scarce specialist resources. It is currently being evaluated in real time as a hybrid service delivery approach aimed at improving access, referral efficiency, and overall system performance in oncology care [15,16,17,18].

To address these limitations, a shift from a purely centralised model to an integrated decentralised–centralised (hub-and-spoke) system has been proposed. This is an initial model that centralises screening, early diagnosis, follow-up, survivorship, and selected treatment services to district and satellite facilities, while centralising high-complexity oncology care at a provincial hub (NMAH). The model is supported by bidirectional referral pathways, task-sharing, telemedicine, and workforce training systems to strengthen continuity and efficiency of care [5,8]. This study was driven by both patient- and health system-level barriers, including late-stage presentation, low health literacy, transport costs, workforce shortages, and geographic inaccessibility [9,10,11,12,13,14]. In response, the decentralised–centralised model aims to improve equity, reduce travel burden, strengthen referral systems, and enhance service efficiency through integrated service delivery and workforce capacity building [15,16,17,18,19,20,21,22,23,24,25,26,27]. This study was conducted to evaluate the implementation process and early impact of the decentralised–centralised (hub-and-spoke) oncology care model as a quality improvement initiative, with specific focus on improving access to care, strengthening referral efficiency, enhancing continuity of treatment, and optimising overall oncology service delivery within a resource-limited rural health system.

## 2. Methods and Materials

### 2.1. Design

This study employed a quantitative quasi-experimental pre–post implementation and quality improvement evaluation design to assess the feasibility and impact of an enriched decentralised–centralised (hub-and-spoke) oncology care model. A pre–post evaluation framework was applied, comparing baseline service-delivery indicators with outcomes observed during the implementation period to evaluate system-level changes over time [10,11].

The study integrated retrospective analysis of routinely collected service utilisation and programme data from April 2023 to February 2025. This period enabled assessment of early implementation trends, service performance, and health system responsiveness. The evaluation focused on changes in access, service integration, and utilisation outcomes following implementation of the decentralised oncology care model. The decentralization component of this study was informed by a previously published protocol assessing the demographic, epidemiological, and clinical profiles of patients receiving cancer care at decentralized oncology services in South Africa, specifically at Nelson Mandela Academic Hospital and Rob Ferreira Hospital. The protocol describes a comparative service-delivery model in which oncology services were shifted from centralized tertiary centers to regional hospitals to improve access to cancer diagnosis, treatment, and follow-up care in underserved provinces [10].

### 2.2. Setting

The study was conducted in a resource-limited rural province in South Africa, served by a single tertiary oncology referral centre (Nelson Mandela Academic Hospital, NMAH) and a network of district hospitals and primary health care (PHC) facilities. The catchment population of approximately three million people is characterised by geographic dispersion, socioeconomic disadvantage, and limited access to specialised oncology services, reflecting constraints typical of low-resource health systems [10,11,12,13].

Old Model: Centralised Oncology Care System

- The previous oncology care model in South Africa, particularly in rural provinces such as the Eastern Cape, was predominantly centralised, with specialised cancer services concentrated in tertiary hospitals such as Nelson Mandela Academic Hospital (NMAH).
- Under this model, screening, diagnosis, treatment, and follow-up care were largely delivered at central referral hospitals, requiring patients to travel long distances from district and primary health care (PHC) facilities.
- Peripheral facilities had limited oncology capacity, with weak diagnostic infrastructure, minimal specialist presence, and poor integration into referral pathways.
- Severe oncology workforce shortages (≈1 oncologist per 3 million population) further constrained service delivery, contributing to delays in diagnosis and treatment initiation.
- The system resulted in fragmented care pathways, high patient travel costs, late-stage presentation, and poor continuity of care, particularly for rural populations.
- Overall, the model was characterised by over-centralisation, inefficiency, and inequitable access to cancer services, especially for underserved communities in LMIC and rural settings [10,11].

New Model: Decentralised–Centralised (Hub-and-Spoke) Oncology Care System

- The new model introduces an integrated decentralised–centralised (hub-and-spoke) oncology care system designed to improve access, efficiency, and continuity of cancer care in resource-limited settings.
- As shown in Figure 1, the model decentralises screening, early diagnosis, follow-up, survivorship, and selected low-risk treatments to district and satellite facilities while maintaining high-complexity oncology care at the central hub (NMAH).
- District and PHC facilities are strengthened through task-sharing, basic diagnostics, trained healthcare workers, and structured referral systems, improving local service-delivery capacity.
- The central hub (NMAH) provides radiotherapy, chemotherapy, complex diagnostics, and inpatient oncology care, while also supporting bidirectional referral pathways and down-referral of stable patients for continuity of care.
- The model is supported by telemedicine, mentorship, training, and operational feedback systems, ensuring integration across all levels of care.
- Overall, the system aims to reduce travel burden, improve early diagnosis, strengthen referral coordination, and optimise scarce oncology workforce resources, aligning with global recommendations for equitable cancer care delivery in LMICs [10,11].

The intervention was structured using a hub-and-spoke implementation framework integrating centralised specialist oncology services with decentralised service-delivery components. Screening, early diagnosis, follow-up care, and selected chemotherapy services were decentralised to district and satellite facilities, while high-complexity oncology care remained centralised at the tertiary hub (NMAH). Key inputs included oncology workforce capacity, clinical infrastructure, standardised treatment protocols, training and mentorship programmes, and supporting health information systems. Core implementation activities included baseline situational analysis, community outreach and screening, referral coordination, workforce training, and patient navigation. The intended outcomes included improved screening uptake, strengthened referral pathways, improved continuity of care, enhanced workforce capacity at peripheral sites, and reduced patient access barriers, contributing to improved efficiency and equity in oncology service delivery [11,14].

### 2.3. Data Collection

Quantitative data were obtained from multiple routine data sources to ensure a comprehensive evaluation of implementation outcomes. Routine Health Information Systems (RHIS) were used to extract data on screening coverage, referral rates, and treatment initiation. Programme monitoring records provided data on outreach activities, workforce training, and patient navigation services. Hospital and clinic records were reviewed to assess oncology service utilisation, treatment continuity, and patient travel burden [14,15].

### 2.4. Data Management

Quantitative data were entered into a secure, password-protected electronic database, with routine validation checks conducted to ensure completeness, consistency, and accuracy before analysis. Data were stored in compliance with institutional data governance requirements to maintain confidentiality and data integrity.

### 2.5. Data Analysis

Quantitative data were analysed using descriptive statistics, including frequencies, percentages, means, and pre–post comparisons of key indicators such as screening coverage, referral completion, patient volumes, workforce capacity, and service uptake. Correlation analysis and linear regression were used to assess relationships between service utilisation trends and implementation variables. Data management and preprocessing were conducted using Microsoft Excel, while statistical analyses were performed using IBM SPSS Statistics version 30) and similarly specify the Stata version Stata 18).

### 2.6. Ethical Considerations

Ethical approval for the study was granted by the Walter Sisulu University Research Ethics Committee (WSU REC-040/2020; 27 August 2022) and Wits University, the relevant provincial health research ethics committee, Nelson Mandela Academic Hospital, and the Provincial Department of Health. All patient-level data were de-identified before analysis to ensure confidentiality and compliance with data protection standards. The study was conducted in accordance with the Declaration of Helsinki, ensuring respect for persons, beneficence, and justice throughout all phases of data collection, analysis, and reporting. The protocol of this study was published elsewhere [10].

## 3. Results

### 3.1. Results (Decentralised Oncology Service Pilot—QI Evaluation)

**Figure 3:**
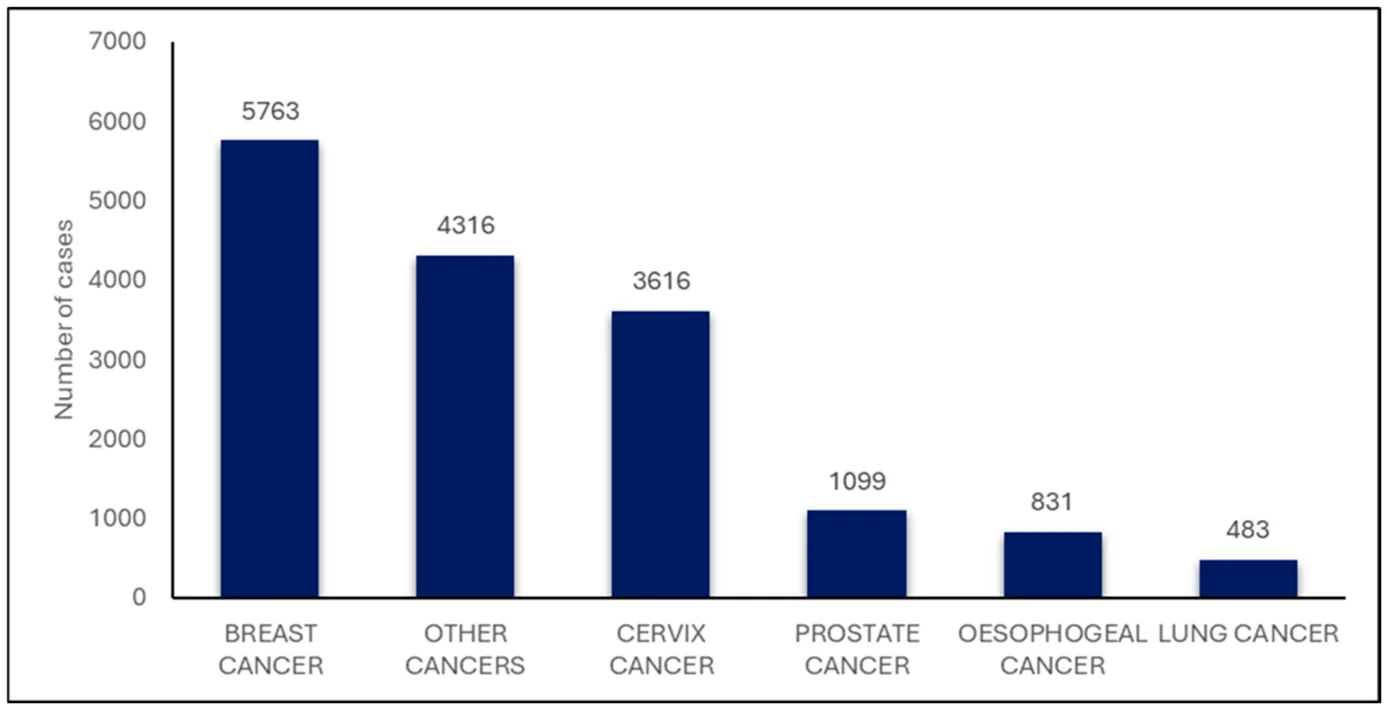
Distribution of cancer cases by type at Nelson Mandela Academic Hospital. April 2023 to February 2025.

Breast cancer (5763) was the most prevalent, followed by other cancers (4316) and cervical cancer (3616). Lower burdens were observed for prostate (1099), oesophageal (831), and lung cancers (483). This distribution highlights the dominance of breast and cervical cancers, indicating priority areas for targeted screening and treatment. Figure 3 provides distribution of cancer cases by type at NMAH.

**Figure 4:**
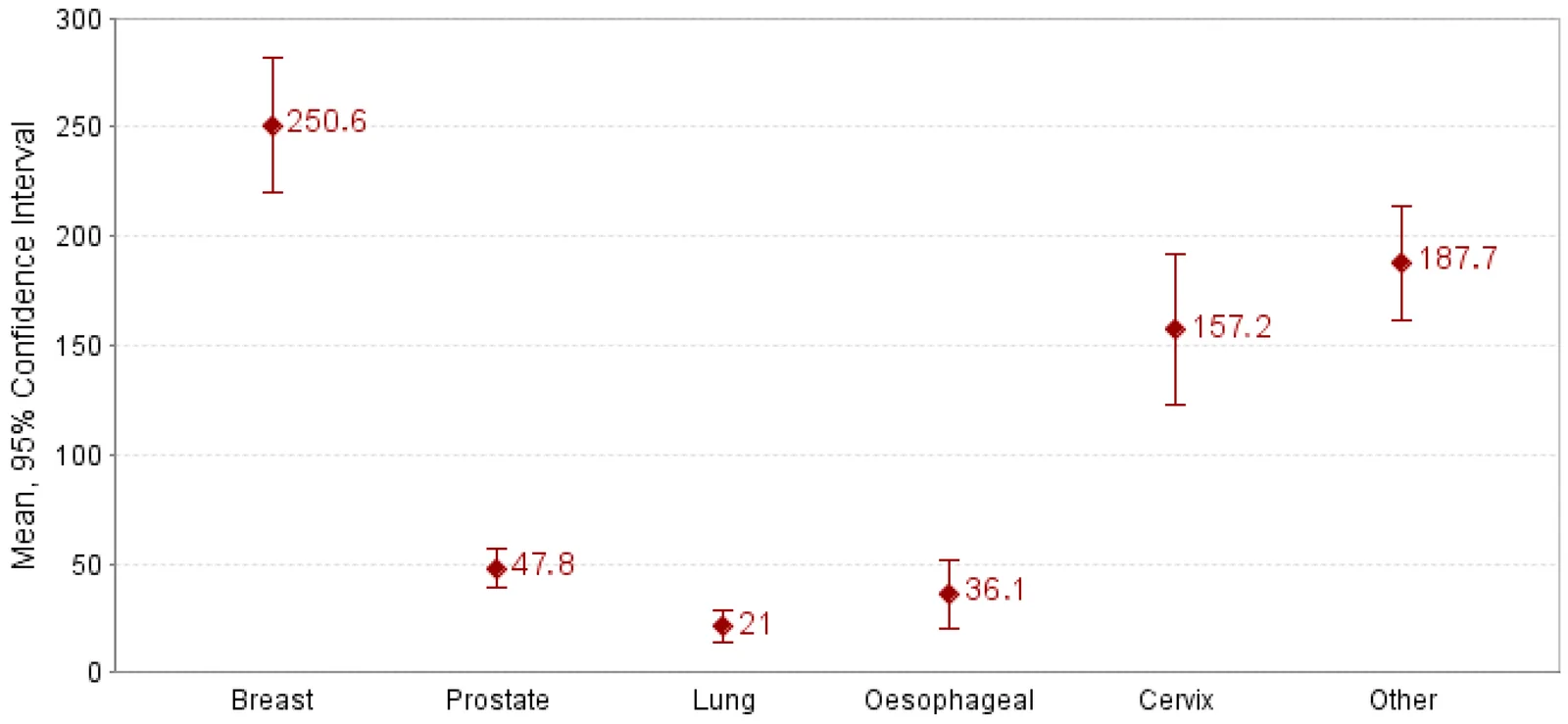
Average monthly cancer cases attended at NMAH with 95% Confidence Intervals at (April 2023–February 2025). Cervical (B = 0.053, *p* = 0.002) and lung cancers (B = 0.224, *p* = 0.007) showed significant increasing trends, while other cancers also increased moderately (*p* = 0.018). Breast cancer showed a non-significant upward trend despite a high burden, and prostate and oesophageal cancers remained stable. These trends indicate a growing and shifting cancer burden requiring targeted prevention strategies.

Breast cancer had the highest monthly mean (250.6), followed by other cancers (187.7) and cervical cancer (157.2), all showing variability (wider CIs). Prostate (47.8), oesophageal (36.1), and lung (21.0) had lower, more stable volumes (narrower CIs). This supports resource allocation aligned with caseload intensity while maintaining coverage for less common cancers. Figure 4 provides the average monthly cancer cases attended at NMAH with 95% Confidence Intervals at (April 2023–February 2025).

Table 1 shows the summary of cancer trends from April 2023 to February 2025. The analysis of cancer trends over 23 months reveals important insights for public health planning.

**Figure 5:**
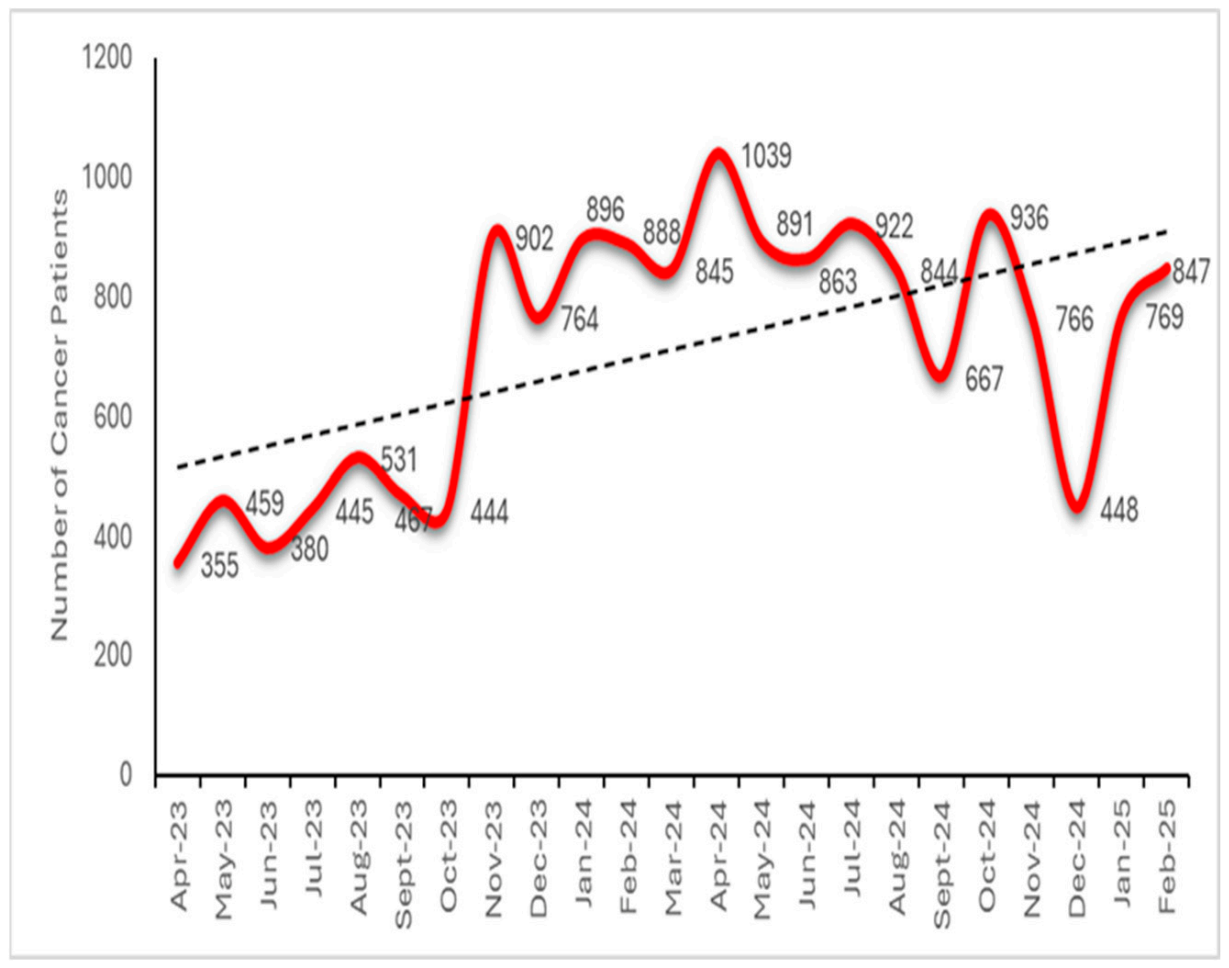
Monthly trend in cancer patient attendance at Nelson Mandela Academic Hospital (April 2023–February 2025).

Patient attendance increased from 355 (April 2023) to 1039 (April 2024) (Figure 5), with a sustained upward trend (B = 18.03, *p* = 0.005), then stabilised at higher levels. Seasonal declines were noted during festive periods. This reflects improved access, decentralised referrals, and increased case detection.

**Figure 6:**
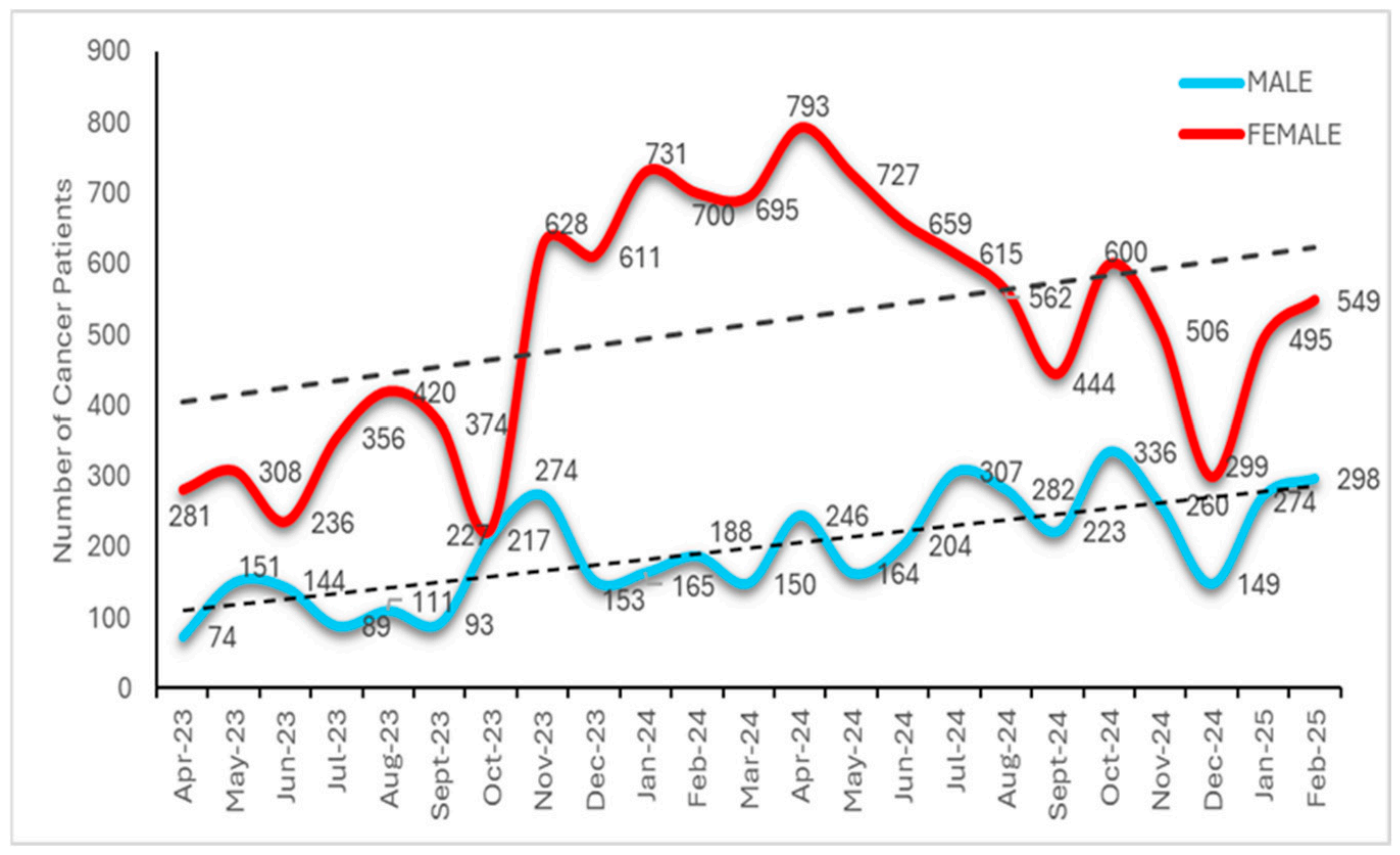
Gender-based trends in cancer patient attendance (April 2023–February 2025).

Female patients consistently accounted for most visits, increasing from 281 to a peak of 793, while male attendance rose steadily from 74 to 298 (B = 8.03, *p* < 0.001 (Figure 6). This indicates improved utilisation overall, but persistent gender disparities.

**Figure 7:**
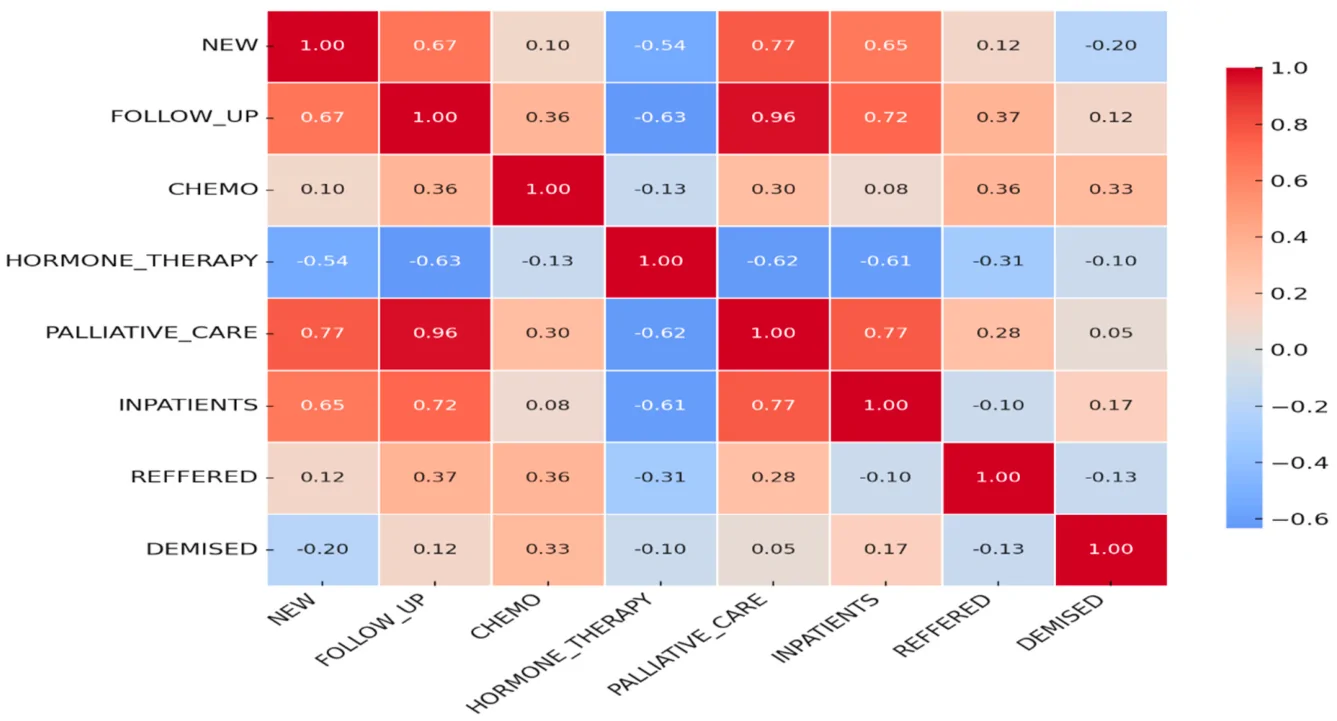
Correlation matrix of service integration indicators.

Strong correlations were observed between follow-up and palliative care (r = 0.965), new cases and palliative care (r = 0.768), and new cases and admissions (r = 0.654). These relationships demonstrate improved integration across the continuum of care with decentralisation. Figure 7 provides the Correlation matrix of service integration indicators.

Key relationships include:

- Strong correlation between new cases and palliative care (r = 0.768);
- Very strong correlation between follow-up and palliative care (r = 0.965);
- Moderate-to-strong correlation between new cases and inpatient admissions (r = 0.654);
- Negative correlation between hormone therapy and high-intensity services.

These findings indicate that as decentralised case detection increased, there was a corresponding rise across the entire care continuum, including follow-up, inpatient care, and palliative services.

Screening coverage increased by 18% (≈38–48% total), and 732,349 individuals were reached through community awareness. Patient travel distance decreased by 56,400 km, and referral pathways improved. Workforce capacity increased with 517 staff trained and 1943 patients supported through navigation. However, gaps remain, screening is below the ≥70% target, diagnostic delays persist, workforce capacity is insufficient, and rural access inequities continue. Continuous quality improvement systems are emerging but not yet fully institutionalised. Across figures and tables, the decentralised model improved access, utilisation, and service integration, with rising patient volumes and strengthened care pathways. Persistent gaps in screening coverage, workforce, and diagnostics highlight priorities for scaling and sustained quality improvement (Table 2).

The implementation of the decentralized–centralized oncology care model demonstrated substantial improvements across service delivery, workforce capacity, and health systems research domains.

#### 3.1.1. Service Delivery Outcomes

Cancer screening coverage for lung, breast, cervical, and AIDS-related cancers increased by 18% within the first year across satellite sites. The program reached 732,349 community members through targeted cancer awareness campaigns, significantly strengthening early detection efforts. In addition, patient access improved substantially, with a 56,400 km reduction in average travel distance, reflecting improved local service availability and decentralization of care. Referral pathways to the central hospital were also strengthened, resulting in more efficient management of patients requiring complex oncology interventions.

#### 3.1.2. Capacity Building Outcomes

A total of 517 health professionals were trained in decentralized oncology care protocols, enhancing clinical competencies across participating facilities. Community health workers were effectively integrated into screening, follow-up, and patient navigation systems, supporting continuity of care for 1943 patients who received structured navigation services.

#### 3.1.3. Operational Research Insights

Operational research identified critical bottlenecks in referral and diagnostic pathways, informing system optimization. Findings provided strong evidence supporting the scalability and sustainability of the hybrid decentralized–centralized oncology care model. Additionally, patient feedback indicated high satisfaction levels and improved continuity of care, reinforcing the acceptability and effectiveness of the intervention. Both genders show a general upward trend, as indicated by the linear trendlines. The number of male patients increased steadily from 74 in April 2023 to 298 in February 2025, with fluctuations along the way. The trendline confirms a modest but statistically observable rise in male patient volume over time.

For female patients, the increase was more pronounced and peaked earlier. The number rose from 281 in April 2023 to a high of 793 in April 2024, before experiencing a notable drop during the festive period (December 2024, January 2025). Despite the fluctuations, the trendline for female patients still points upward, suggesting a stronger long-term growth pattern compared to their male counterparts.

Notable fluctuations are visible for both genders. For males, the peaks occur in November 2023 (274) and October 2024 (336), whereas female patient volumes show more dramatic surges and declines, particularly around November 2023 to April 2024 and again in December 2024.

## 4. Discussion

This study showed that an integrated hub-and-spoke model for oncology care can significantly enhance cancer service delivery in a rural, resource-limited health system. After implementation, there was a noticeable improvement in screening uptake, more efficient referral processes, reduced patient travel distances, and increased community involvement in cancer awareness initiatives [18,19].

The intervention also contributed to stronger health system capacity through structured training, mentorship, and task-sharing between oncology specialists and primary healthcare providers, aligning with global recommendations for expanding the cancer care workforce [20]. Furthermore, improvements were observed in continuity of care, patient acceptance of services, and the identification of operational gaps within the care pathway.

In South Africa, oncology services remain largely concentrated in urban tertiary hospitals, resulting in delayed diagnosis, long waiting times, and unequal access for rural populations [21].

Although provinces such as Gauteng and Western Cape have implemented decentralisation efforts, these initiatives are often limited in scope, fragmented, and focused on specific cancer types rather than integrated care pathways. In contrast, the model evaluated in this study establishes a coordinated system that connects community, district, and tertiary levels through formal referral structures, governance mechanisms, and ongoing feedback systems. This represents a more integrated and system-wide approach compared with current provincial practices.

Across sub-Saharan Africa, oncology care systems face similar structural challenges, including shortages of trained specialists, limited diagnostic resources, and frequent late-stage presentation of cancer [18]. While several countries, such as Kenya, Uganda, and Malawi, have introduced decentralised cancer screening programmes, these are mostly vertical interventions focusing on single diseases, particularly cervical cancer.

Unlike these fragmented approaches, the model in this study incorporates the full continuum of cancer care, including prevention, early detection, diagnosis support, treatment coordination, survivorship, and palliative care. This comprehensive integration offers a more sustainable and system-oriented approach suitable for low-resource African settings.

In high-income countries such as Australia, Canada, and several European nations, hub-and-spoke oncology systems are well established and supported by strong infrastructure, advanced technology, and a high density of oncology specialists [22,23]. These systems allow efficient coordination between primary care providers and specialised cancer centres.

Although the structural design is similar, the main difference lies in resource availability and technological support. This study demonstrates that comparable functional outcomes can still be achieved in resource-constrained environments through workforce development, task-sharing, and effective use of existing health infrastructure rather than dependence on advanced digital systems [20].

### 4.1. Strengths of the Study

A major strength of this study lies in its evaluation of a decentralised–centralised oncology model implemented under real-world constraints in a resource-limited rural health system. Rather than a controlled experimental setting, the model was assessed within routine service delivery, enhancing its external validity, contextual relevance, and transferability to similar low- and middle-income settings [24]. This allows for a more realistic understanding of how oncology service redesign performs under conditions of workforce shortages, infrastructural limitations, and high patient demand.

A further strength is that the intervention is embedded within a health system strengthening and service-delivery redesign framework, rather than a stand-alone clinical intervention. The model leverages existing infrastructure and prioritises task-sharing, referral optimisation, and capacity building, reducing reliance on additional specialist recruitment. This enhances both feasibility and sustainability in rural oncology care contexts.

In addition, the implementation process was iterative, supported by continuous feedback from frontline healthcare workers, community-based providers, and referral facilities. This adaptive learning approach strengthened operational integration, improved referral coordination, and enhanced responsiveness of the system over time, thereby increasing the practical robustness of the model.

### 4.2. Limitations

This study has several limitations that should be considered when interpreting the findings. First, the before-and-after design without a control group limits causal inference, as observed changes in service indicators may be influenced by concurrent health system reforms, policy shifts, or contextual changes unrelated to the intervention.

Second, the study relies on routine health information systems, which may introduce variability in data completeness, reporting accuracy, and consistency across decentralised sites with differing data management capacity. This may affect the precision of some measured outcomes.

Third, important clinical variables were not included. Cancer staging and other biomedical indicators, such as disease severity at presentation, progression, and treatment response, were not systematically captured, limiting interpretation of whether increased case detection reflects earlier diagnosis or a higher burden of advanced disease.

In addition, patient-reported outcomes, including quality of life (QoL), psychosocial wellbeing, and functional status, were not assessed. This limits understanding of the patient-centred impact of decentralised oncology care beyond service utilisation and access indicators.

Finally, the relatively short evaluation period limits the conclusions that can be drawn regarding long-term outcomes such as survival, mortality, and sustained system performance. As a process evaluation of an evolving implementation model, some operational challenges—particularly diagnostic turnaround times—remain unresolved.

### 4.3. Clinical and Health System Implications

The findings suggest that selected components of oncology care can be safely decentralised when supported by structured clinical protocols, clear referral pathways, and strong governance systems. In this model, district and primary health care facilities are best positioned to deliver cancer awareness, screening, initial assessment, follow-up, and supportive care. At the same time, surgical oncology and complex treatments remain appropriately centralised at the tertiary level (Nelson Mandela Academic Hospital). The model demonstrates potential to improve early engagement with care, reduce delays between diagnosis and treatment initiation, and strengthen continuity across levels of care. It also highlights the importance of multi-cadre workforce development—including clinicians, nurses, community health workers, medical physicists, and data personnel—to support safe task-sharing and effective referral coordination.

From a health system perspective, the study supports integration of oncology services into primary health care platforms as part of broader system strengthening. However, effective decentralisation requires continuous supervision, diagnostic capacity strengthening, and well-functioning referral networks to ensure safety, equity, and quality of care.

### 4.4. Future Directions and Expanded Implementation Priorities

Future implementation should incorporate strengthened digital health systems, including tele-oncology platforms, electronic referral tracking, and real-time patient monitoring, to address persistent bottlenecks such as diagnostic turnaround delays and referral inefficiencies. A key priority for future phases is the integration of more comprehensive evaluation frameworks. This includes systematic collection of cancer staging data and other biomedical indicators to enable clearer differentiation between early detection and late-stage presentation patterns. In addition, future studies should incorporate validated patient-reported outcome measures, including health-related quality of life (QoL), symptom burden, and psychosocial wellbeing, to ensure a more patient-centred evaluation of decentralised oncology care. Importantly, future implementation should also reintegrate qualitative methods to capture the lived experiences of patients, healthcare workers, and system stakeholders, which are critical for understanding acceptability, feasibility, and contextual drivers of implementation success [25,26,27].

Finally, longitudinal and multicentre expansion studies are needed to evaluate survival outcomes, cost-effectiveness, and scalability across other cancer types and rural districts. This will strengthen the evidence base for decentralised oncology care as a sustainable model for resource-limited health systems.

## 5. Conclusions

The hub-and-spoke model for oncology care represents a practical and scalable approach for improving cancer services in rural and resource-constrained settings. By combining decentralised primary care delivery with centralised specialist support, the model improves access, strengthens system efficiency, and enhances continuity of care. When compared with existing approaches in South Africa, other African countries, and high-income settings, the findings indicate that effective oncology system strengthening is not solely dependent on resources, but also on how services are organised, coordinated, and supported. Overall, this model provides a sustainable strategy for reducing disparities in cancer care and improving outcomes in underserved populations.

## Figures and Tables

**Table 1:** Summary of simple linear regression analyses for trends in cancer cases (April 2023–February 2025).

Cancer Type	Pearson Correlation (r)	R^2^	B Coefficient	*p*-Value	95% CI for B	Interpretation
Breast	0.301	0.091	0.028	0.163	[−0.012, 0.069]	Positive but not statistically significant trend
Prostate	0.164	0.027	0.055	0.455	[−0.096, 0.207]	No significant trend
Lung	0.549	0.302	0.224	0.007	[0.069, 0.379]	Significant increasing trend
Oesophageal	0.085	0.007	0.016	0.701	[−0.068, 0.099]	No significant trend
Cervical	0.619	0.383	0.053	0.002	[0.022, 0.083]	Strong and significant increasing trend
Other cancers	0.49	0.24	0.054	0.018	[0.010, 0.098]	Moderate and statistically significant trend

**Table 2:** Eastern Cape Cancer Services—QI Evaluation Against WHO Targets.

Domain	Indicator	Initial (Baseline—Eastern Cape)	Achieved	WHO Target	Gap to Achieve Target	QI Interpretation
Service Delivery	Screening coverage (breast, cervical, lung, HIV-related cancers)	~20–30% population coverage	+18% increase (≈38–48% total est.)	≥70% screening coverage (cervical: 70% by age 35 and 45)	~22–32% below target	Good progress, but still far from population-level impact
Service Delivery	Community reach	Limited, fragmented outreach	732,349 individuals reached	≥80–90% population awareness	Likely still below full provincial coverage	Strong scale-up; needs sustained and repeated engagement
Service Delivery	Patient access (distance to care)	Highly centralized services; long travel burden	56,400 km reduction	Care available within 2 h travel time	Some rural populations are still underserved	Decentralization is working, but geographic inequity remains
Service Delivery	Referral efficiency	Delayed, weak referral pathways	Improved referral flow	≤2 weeks referral-to-diagnosis time	Likely still delays in diagnostics	System improved, but diagnostic turnaround is still a bottleneck
Capacity Building	Health workforce trained	Severe oncology workforce shortage	517 trained	Adequate density (WHO recommends ~4.45 health workers/1000 population overall)	Still below the required oncology workforce density	Training improved capacity, but the workforce gap remains large
Capacity Building	CHW integration	Minimal structured navigation support	1943 patients supported	Full integration of CHWs in the continuum of care	Coverage still limited relative to need	Strong model, needs scale-up across districts
Capacity Building	Continuity of care	Weak follow-up, high loss to follow-up	Improved tracking systems	≥90% treatment completion and retention	Likely still below optimal retention	Digital/structured tracking improved outcomes
Operational Research	System bottlenecks	Poorly documented system inefficiencies	Bottlenecks identified and addressed	Continuous quality improvement systems in place	CQI is not yet fully institutionalized	Shift toward data-driven decision-making achieved
Operational Research	Model effectiveness	No validated decentralization model	Evidence supports scalability	National/provincial scale-up	Needs policy adoption and funding	Strong evidence base for expansion
Operational Research	Patient experience	Limited patient-centred evaluation	High satisfaction reported	Patient-centred care integrated into systems	Needs routine measurement tools	

## Data Availability

The datasets generated and analyzed during the current study are available from the corresponding author on reasonable request, subject to institutional data-sharing policies and ethical approval.

## Abbreviations

ASCO	American Society of Clinical Oncology
CHW	Community Health Worker
HIV	Human Immunodeficiency Virus
LMIC	Low- and Middle-Income Countries
NMAH	National/Provincial Oncology Referral Hospital (Hub)
PHC	Primary Health Care
RHIS	Routine Health Information System
WHO	World Health Organization
